# The effects of aroma inhalation on the quality of sleep, professional quality of life, and near-misses in medication errors among emergency room nurses on night duty in Korea: a randomized controlled trial

**DOI:** 10.7586/jkbns.24.040

**Published:** 2025-02-19

**Authors:** Jungha Son, Chul-Gyu Kim

**Affiliations:** 1 Chungbuk Emergency Medical Support Center, National Emergency Medical Center, National Medical Center, Cheongju, Korea; 2 Department of Nursing Science, Chungbuk National University, Cheongju, Korea

**Keywords:** Aromatherapy, Sleep, Quality of life, Medication errors

## Abstract

**Purpose:**

This study investigated the effects of aroma inhalation on sleep quality, professional quality of life (QoL), and near-misses in medication errors during night shifts among emergency room nurses.

**Methods:**

A randomized crossover experimental design was used to determine the effects of this intervention. The research participants included 55 nurses (29 in Group 1 and 26 in Group 2) who worked as nurses in the emergency room at a tertiary general hospital in Chungcheongbuk-do, South Korea. Aroma inhalation was conducted on the night shift. Sleep quality, professional QoL, and near-misses in medication were measured before and after inhalation of the aroma. Data was analyzed using the independent t-test, the chi-square test, and a linear mixed-effects model.

**Results:**

The aroma treatment group had significantly better sleep quality than the non-treatment group (*p* < .010), and the sleep time on the third day of aroma treatment was longer than that of the non-treatment group (*p* = .008). However, there were no signs of improvement in professional QoL or near-misses in medication errors in response to aroma treatment.

**Conclusion:**

Aroma inhalation effectively improved sleep quality and increased sleep duration in emergency room nurses. Therefore, aroma inhalation is suggested as an intervention to improve the sleep quality of emergency room nurses who work night shifts. Follow-up studies are needed to build a more robust evidence base to inform strategies for improving nurses' professional QoL and patient safety during medication management.

## INTRODUCTION

### 1. Background

Approximately 90% of clinical nurses work shifts [1], which disrupt their natural sleep-wake cycle and lead to physical and psychological health issues, such as sleep disorders [2], decreased empathy satisfaction, and reduced professional quality of life (QoL) due to burnout [3]. Shift work disrupts circadian rhythms, reduces sleep quality, decreases total sleep duration during night shifts to an average range of 4.98 to 5.86 hours [4], and causes sleep disturbances in nurses [5], reducing accuracy and efficiency and increasing patient safety incidents, such as needlestick injuries and medication errors [2]. Previous studies have highlighted the significant effect of sleep duration on medication errors among nurses [6]. Poor sleep quality is associated with an increased likelihood of near misses in medication administration [7], and night shift work contributes to medication administration errors [8]. Particularly, emergency room nurses have poorer sleep quality than nurses in other departments [2], which has serious consequences for emergency patients who require immediate treatment [9].

Professional QoL for individuals in helping professions refers to their subjective QoL [3], characterized by compassion satisfaction and fatigue; the latter is further divided into burnout and secondary traumatic stress. Nurses' compassion satisfaction contributes to enhanced nursing performance and professional competence [10]; compassion fatigue leads to increased nursing errors and deteriorated quality of nursing services due to emotional exhaustion and a sense of powerlessness [3]. Burnout among nurses increases the likelihood of medical accidents due to factors including reduced job performance. Secondary traumatic stress among emergency room nurses leads to sleep problems and anxiety [11]. Emergency room nurses frequently exposed to trauma and violence experience severe compassion fatigue in 86% of cases, and have lower compassion satisfaction [11]. Therefore, efforts are needed to enhance their professional QoL by improving compassion satisfaction and reducing compassion fatigue. Recent studies have highlighted the intricate relationship between nurses' professional QOL and patient safety activities, including medication management [7]. The findings indicate a positive correlation between compassion satisfaction and patient safety activities and a negative correlation between compassion fatigue and patient safety activities. Jeanmonod et al. [12] further emphasized the impact of compassion fatigue on patient care quality. They indicated that increased compassion fatigue among health professionals can lead to reduced quality of care provided to patients.

Sleep disorders and poor professional QoL contribute to medication errors among nurses [13,14]. Therefore, medication errors can be reduced by improving the sleep quality and professional QoL of emergency room nurses. Emergency rooms, which frequently use high-risk medications such as anticoagulants, insulin, and high-concentration electrolytes, face a heightened risk of medication errors [13]. This concern is underscored by medication-related errors comprising 43% of the reported incidents in the 2022 Patient Safety Statistics Annual Report [15]. Recognizing and managing near misses—incidents that could have resulted in problems but did not—are crucial for preventing major accidents and avoiding harmful medication errors [16]. Given the interrelation between sleep disorders, reduced professional QoL, and safety issues such as medication errors among emergency room nurses [17], effective interventions are essential to address these significant concerns related to patient care duties.

Pharmacotherapy is commonly used to manage emotional disorders and address sleep disturbances. However, prolonged pharmacological treatments can lead to physical side effects and drug resistance. This has led to an increased interest in non-pharmacological alternatives [18]. While many complementary and alternative therapies have demonstrated effectiveness, they often face limitations, such as dependence on specific locations, significant time requirements, and need for administration by trained practitioners, making self-administration challenging. In contrast, aromatherapy offers several advantages, including flexibility in terms of location and time, cost-effectiveness, minimal side effects, and a lack of systemic accumulation, while producing relatively rapid therapeutic effects [19]. Aromatherapy uses essential oils extracted from various plant parts, with different oils offering benefits such as relieving fatigue, anxiety, depression, stress, and insomnia and providing antibacterial effects [18]. These oils are safe because they are metabolized and excreted by the body without accumulation [19]. Studies on aromatherapy for emotional disorders found lavender to be the most commonly used essential oil (27.6%), followed by bergamot (7.4%) and ylang-ylang (6.8%) [20]. For sleep disorders, lavender was the most frequently used oil (50%) [21]. The human brain responds to stress by secreting neurotransmitters, including acetylcholine, dopamine, and serotonin. Serotonin is particularly effective in relaxation owing to its similarity to tranquilizers produced in the brain. Oils rich in esters, such as lavender, promote the release of serotonin, thereby alleviating stress, insomnia, and anxiety, as well as having relaxing and calming effects [19]. Bergamot oil has a balancing effect on hypothalamic activity and acts on the central nervous system to simultaneously uplift mood and provide calming relaxation [22]. Ylang-ylang oil influences the nervous system to alleviate emotions, such as anger and anxiety, provide comfort and happiness, and is used as a relaxant or sedative because of its calming effects on nervousness and insomnia [23]. Aromatic oils are categorized into three classes based on volatility and fragrance characteristics: top notes, which evaporate quickly and last less than three hours; middle notes, which have moderate evaporation rates and persist for six hours to two or three days; and base notes, which linger for the longest and last over seven days. Blending these notes in appropriate proportions yields a synergistic effect. Bergamot and lavender were classified as the top notes, ylang-ylang as the middle notes, and sandalwood as the base notes [24].

Inhalation is the most effective aromatherapy method [25], offering benefits in terms of duration, ease of use, and applicability without special training [19]. Aroma inhalation can stimulate the brain within 0.1 seconds of exposure to essential oils, directly impacting the central nervous system and eliciting the fastest response among aromatherapy methods [26]. Upon inhalation, essential oils are processed by the olfactory system, reaching the cerebral cortex, hypothalamus, and ultimately, the pituitary gland. This sequence affects the autonomic nervous system, suppressing adrenaline secretion [27]. As the process continues, aromatic molecules are inhaled into the lungs, reaching the bronchi and alveoli, where they enter the bloodstream, and their effects are disseminated throughout the body [18]. Aroma inhalation benefits sleep, professional QoL, and anxiety among emergency department and night-shift nurses, even with short intervention periods [28-31]. This method effectively increased the professional QoL among emergency room nurses when patchouli oil was applied twice for 20 min at approximately 10 PM after their afternoon shift [28]. Additionally, sleep quality improved when lavender essential oil inhalation was provided to shift-working nurses for three days during sleep—starting before bedtime after night shifts and continuing until seven hours after sleep onset [29]. Improved sleep quality was also noted when blended essential oils (mandarin, sweet marjoram, frankincense, and Roman chamomile blended in a ratio of 1:1:1:1) were inhaled by nurses working fixed night shifts for three days [30]. Anxiety was reduced in health workers, including nurses in the emergency department, after inhaling lavender aromatherapy for 10 min [31]. Despite these positive effects, research is lacking on the impact of aroma inhalation on emergency room nurses, who face unique challenges such as poor sleep quality and an increased risk of medication errors. Therefore, this study aimed to investigate the effects of aroma inhalation on sleep quality, professional QoL, and near-misses related to medication errors among emergency room nurses.

### 2. Hypotheses

Hypothesis 1: “Exposure to aroma inhalation improves sleep quality.”

· Sub-hypothesis 1-1: “Exposure to aroma inhalation enhances sleep quality.”

· Sub-hypothesis 1-2: “Exposure to aroma inhalation increases sleep duration.”

Hypothesis 2: “Exposure to aroma inhalation increases professional QoL.”

· Sub-hypothesis 2-1: “Exposure to aroma inhalation increases compassion satisfaction.”

· Sub-hypothesis 2-2: “Exposure to aroma inhalation decreases compassion fatigue.”

Hypothesis 3: “Exposure to aroma inhalation reduces near misses in medication errors.”

## METHODS

### 1. Study design

**Figure 1.**
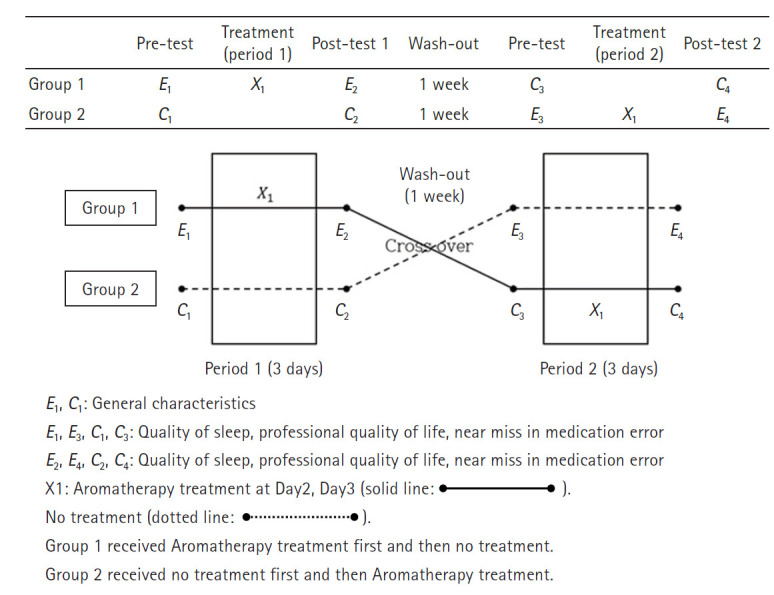
Crossover design.

Using a randomized crossover experimental study design, this study examined the effect of aroma inhalation on sleep quality, professional QoL, and near misses in medication errors among emergency room nurses on night duty (Figure 1).

### 2. Setting and sample

The study participants were nurses working rotating shifts at a tertiary-level general hospital emergency room in Chungcheongbuk-do who consented to participate. The inclusion criteria were individuals working in a three-shift rotation; without cardiovascular, cerebrovascular, or kidney-related diseases; and with normal olfactory function. Individuals who consumed alcohol during the study period, took sedatives, anxiolytics, or sleep-inducing medications, or had neurological or psychiatric disorders were excluded.

The sample size was determined according to the sample size calculation formula of Chow and Liu [32] for a 2 × 2 crossover design difference test with a statistical power of 90%, significance level of .05, and effect size and standard deviation based on Kim and Hur [29]. The minimum sample size calculated for a 2 × 2 crossover design was one, resulting in 24 participants. Considering potential dropouts, a total of 60 emergency room nurses working night-shifts at Chungbuk National University Hospital were recruited, as none met the exclusion criteria. All participants were assigned unique identification numbers and randomly allocated to Group 1 (aroma inhalation -> no treatment) or Group 2 (no treatment -> aroma inhalation) using the R 4.1.0 program with a 1:1 allocation ratio. Due to the nature of the study, blinding was not feasible, and participants were aware of their group assignments. One participant from Group 1 (resigned) and four from Group 2 (pregnancy, use of psychoactive drugs, relocation, and unwillingness to participate) were drop out. Thus, the final analysis included 29 participants in Group 1 and 26 in Group 2, for a total of 55 participants, meeting the required sample size.

### 3. Instruments

The following instruments were used after obtaining permission from the authors.

#### 1) Sleep quality

##### (1) Sleep quality

The Verran Snyder-Halpern (VSH) sleep scale, developed by Verran and Snyder-Halpern [33], was measured using the Korean version adapted by Kim and Kang [34]. This instrument consisted of eight items. Each item is scored on a scale of 0 to 10, with a total score ranging from 0 to 100. Higher scores indicated better sleep quality. The reliability of Cronbach's α was .82 in Kim and Kang [34], and .87 in this study.

##### (2) Sleep time

Sleep time (in minutes) was measured using a self-reporting method. On day 1, the participants reported their total sleep time from the previous day of nighttime sleep and daytime sleep before their shift. For days 2 and 3, the participants reported their sleep duration after completing their night shifts [35].

#### 2) Professional QoL

The Korean version of the Professional Quality of Life Scale–Ver 5, an open-access instrument developed by Stamm [3], was adapted by Kim and Choi [36]. It consists of 10 items on compassion satisfaction and 20 on compassion fatigue. Each item is rated on a 5-point Likert scale. The total score ranges from 10 to 50 points for compassion satisfaction and 20 to 100 points for compassion fatigue. Higher scores indicate higher levels of compassion satisfaction and more severe compassion fatigue. The reliability of Cronbach's α was reported as .88 for compassion satisfaction and .81 for compassion fatigue by Stamm [3], and .89 for compassion satisfaction and .76 for compassion fatigue by Kim and Choi [36]. The reliability was .92 for compassion satisfaction and .76 for compassion fatigue in this study.

#### 3) Near misses in medication errors

A near miss in the medication tool was employed, adapted, and refined based on that originally developed by Park and Lee [37]. Park and Lee's tool comprises nine items designed to assess near-miss medication errors among ward nurses. However, one item (inaccurately transcribing physicians’ orders onto the medication card or handover record) was considered unsuitable for emergency room scenarios and was therefore excluded, leaving eight items. The modified tool, validated for content validity by five experts, involved removing two items with a Content Validity Index below 0.8 (administering medication to the patient more than one hour earlier than prescribed and administering a different formulation than that prescribed by the doctor) and the addition of one item (administering medication to a different patient), resulting in a total of seven items. This instrument assigns one point to each occurrence of an item, with a maximum of five points for five or more occurrences. The total score ranges from 0 to 35 points. Higher scores indicate a greater number of near-misses. While the tool demonstrated a Cronbach's α of .77 during its development [37], it exhibited a reliability of .59 in this study.

### 4. Study intervention

Based on consultations with two aromatherapy experts and prior research [38], the aromatherapy methods were selected. Bergamot (top note), effective for calming and relaxation; lavender (middle note), for alleviating insomnia and promoting relaxation; and ylang-ylang (middle note), for feelings of comfort and happiness and effective against nervousness and insomnia, were selected. The essential oils of lavender, bergamot, and ylang-ylang were blended in a ratio of 3:2:1 [38] before the experimental intervention. The aroma inhalation technique was implemented with five emergency room nurses to evaluate their scent preferences, identify precautions, determine inhalation durations, and establish the distance of the aroma stone application, thus addressing any potential discomfort or issues that could arise during the intervention.

Aroma inhalation was administered using a glass bottle containing 2 cc of the oil mixture (lavender, bergamot, and ylang-ylang in a 3:2:1 ratio) and a dropper. Two drops of the oil were applied to an aroma stone before bedtime and placed within 30 cm of their head, allowing continuous inhalation of the fragrance during sleep. The aromatherapy intervention was administered over two consecutive night shifts. In the morning following the first night shift (day 2) and the morning following the second night shift (day 3), participants were instructed to sleep within two hours of finishing their night shifts, allowing aroma inhalation for seven hours during their sleep. The non-application of aroma inhalation followed the same schedule, with participants instructed to sleep within two hours after their morning on the day following the start of their night duty (day 2) and the subsequent day (day 3), but without aroma inhalation. To prevent carry-over effects, a one-week washout period was implemented between experimental treatments. Specifically, Group 1 received aromatherapy for two days, followed by a one-week washout period, and then two days without aromatherapy. Group 2, on the other hand, had two days without aromatherapy, followed by a one-week washout period, and then two days with aromatherapy (Figure 1).

### 5. Data collection

**Figure 2.**
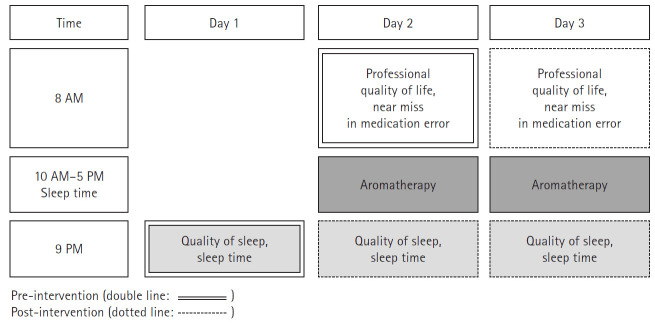
Pre- and post-intervention measurements.

The data collection period was from July 20, 2021, to October 19, 2021. Data was collected from nurses working in the emergency room of Chungbuk National University Hospital who agreed to participate in the study and understood its purpose, significance, and research procedures. The timing of data collection was determined based on previous studies [29]. Data was collected using self-administered questionnaires. Researcher conducted pre-assessments in the evening of the first day (day 1) and the morning of the second day (day 2). Sleep quality was measured at 9:00 PM, before the start of the first night shift (day 1). Professional QoL and near-misses in medication errors were assessed at 8:00 AM, following completion of the first night shift (day 2). Researcher conducted post-assessments on the evenings of the second and third days (days 2 and 3) and the morning of the third day (day 3). Sleep quality was measured at 9:00 PM before the start of the second night shift (day 2) and at 9:00 PM on the day following the completion of the night shift (day 3). Professional QoL and near-misses in medication errors were assessed at 8:00 AM, following the completion of the second night shift (day 3) (Figure 2).

### 6. Data analysis

Data were analyzed using SPSS version 25.0 (IBM Corp., Armonk, NY, USA) and R version 4.1.1 (R Foundation for Statistical Computing, Vienna, Austria). The general characteristics of the participants were analyzed using descriptive statistics, including frequencies, percentages, means, and standard deviations. Homogeneity was tested using independent t-tests and χ² tests. To assess the effects of aroma inhalation, pretreatment homogeneity, and its effects on sleep, professional QoL, and near misses in medication errors were examined using a crossover trial linear mixed effects model. The reliability of the measurement tools was assessed using Cronbach's coefficient.

The following statistical model was used for the analysis.


Y∼Group(Subject)+Period+Treatment+ε

Y: dependent variable (sleep quality, professional QoL, and near miss medication); Group: sequence group, RT or TR; RT: reference (no) treatment at period 1, test treatment at period 2; TR: test treatment at period 1, reference (no) treatment at period 2; Subject: subject is nested in the sequence group, expressing inter-individual random variability; Period: 1 or 2; Treatment: T (test, aroma therapy) or R (reference, no treatment); ε: error

### 7. Ethical considerations

The Institutional Review Board (IRB) at Chungbuk National University Hospital approved the research through ethical review (IRB No. CBNUH2021-02-030-001) on April 1, 2021. This study was registered with the Clinical Research Information Service under the Korea Disease Control and Prevention Agency (KCT0009874). Written informed consent was voluntarily obtained from all participants after they were thoroughly informed about the purpose and procedures of the study. The participants were informed of their right to stop participating at any time if they did not wish to do so and that there would be no disadvantages. To protect participants’ privacy, a unique ID code was assigned to each participant, and the collected data were analyzed according to personal information processing guidelines and stored on a locked computer for three years before being discarded. This study adhered to the reporting guidelines of the Consolidated Standards of Reporting Trials.

## RESULTS

### 1. Homogeneity of general characteristics of participants and dependent variables

There were no statistically significant differences in general characteristics between Group 1 and Group 2 (*p* > .05, Table 1). Pre-measurement values of sleep quality, professional QoL, and near-misses in medication errors did not show significant differences based on group or period (*p* > .05, Table 2).

### 2. Hypotheses test

Hypothesis 1: the hypothesis that "Exposure to aroma inhalation improves sleep quality" is examined in detail through the following sub-hypotheses.

Sub-hypothesis 1-1: the hypothesis that "exposure to aroma inhalation enhances sleep quality" was supported (Table 3). On day 2, sleep quality significantly increased when aroma inhalation was applied (49.28 ± 13.23) compared to no treatment (43.10 ± 14.27), with statistical significance (t = 3.04, *p* = .004, 95% confidence interval [CI] = 2.10~10.26). On day 2, in Group 1, sleep quality increased with aroma inhalation (49.95 ± 12.89) compared to no treatment (43.71 ± 15.51); in Group 2, sleep quality increased with aroma inhalation (48.54 ± 13.82) compared to no treatment (42.42 ± 13.02). The sleep quality on day 3 significantly improved when aroma inhalation was applied compared to no treatment, with statistical significance (t = 3.64, *p* = .001, 95% CI = 3.03~10.43), with scores of 53.85 ± 13.35 for aroma inhalation and 47.11 ± 12.64 for no treatment. On day 3, in Group 1, the sleep quality increased with aroma inhalation (54.48 ± 14.83) compared to no treatment (47.48 ± 13.07). In Group 2, the sleep quality also increased with aroma inhalation (53.15 ± 11.74) compared to no treatment (46.69 ± 12.39).

Sub-hypothesis 1-2: the hypothesis that "Exposure to aroma inhalation increases sleep duration" was partially supported (Table 3). On day 2, sleep duration increased after aroma inhalation (6.64 ± 1.72) compared to no treatment (6.49 ± 1.65), although the difference was not statistically significant (t = 0.60, *p* = .552, 95% CI = −0.34~0.63). However, in Group 1, sleep duration increased after aroma inhalation (6.60 ± 1.09) compared to no treatment (6.43 ± 1.90). In Group 2, sleep duration also increased after aroma inhalation (6.67 ± 1.28) compared to no treatment (6.56 ± 1.34). On day 3, sleep duration significantly increased after aroma inhalation (6.71 ± 1.84) compared to no treatment (5.93 ± 1.66), with statistical significance (t = 2.76, *p* = .008, 95% CI = 0.21~1.34). On day 3, in Group 1, sleep duration increased after aroma inhalation (6.66 ± 2.09) compared to no treatment (5.89 ± 1.71). In Group 2, sleep duration also increased after aroma inhalation (6.77 ± 1.56) compared to no treatment (5.98 ± 1.63).

Hypothesis 2: the hypothesis that “Exposure to aroma inhalation increases professional QoL” is examined in detail using the following sub-hypotheses:

Sub-Hypothesis 2-1: the hypothesis that “Exposure to aroma inhalation increases compassion satisfaction” was not supported (Table 3). Compassion satisfaction increased after aroma inhalation (30.60 ± 5.95) compared to no treatment (30.15 ± 5.95), but this difference was not statistically significant (t = 0.99, *p* = .326, 95% CI = −0.46~1.36). In Group 1, compassion satisfaction slightly increased from no treatment (30.07 ± 4.68) to after aroma inhalation (30.62 ± 4.76), while in Group 2, it also slightly increased from no treatment (30.23 ± 7.20) to after aroma inhalation (30.58 ± 7.15).

Sub-hypothesis 2-2: the hypothesis that “Exposure to aroma inhalation decreases compassion fatigue” was not supported (Table 3). Compassion fatigue slightly decreased after aroma inhalation (55.58 ± 7.38) compared to no treatment (56.36 ± 7.10), but this difference was not statistically significant (t = −1.44, *p* = .156, 95% CI = −1.98~0.33). Group 1 showed no change in compassion fatigue between no treatment (55.00 ± 6.77) and aroma inhalation (55.00 ± 7.49). However, Group 2 showed decreased compassion fatigue after aroma inhalation (56.23 ± 7.34) compared to no treatment (57.88 ± 7.27).

Hypothesis 3: the hypothesis that “Exposure to aroma inhalation reduces near misses in medication errors” was not supported (Table 3). Near misses in medication error were 0.07 ± 0.33 points during no treatment and 0.00 ± 0.00 points after aroma inhalation; this difference was not statistically significant (t = −1.80, *p* = .080, 95% CI = −0.16~0.01). In Group 1, near misses in medication error were 0.00 ± 0.00 points both during no treatment and after aroma inhalation. In Group 2, near miss in medication error was 0.15 ± 0.46 points during no treatment and 0.00 ± 0.00 points after aroma inhalation.

## DISCUSSION

This study examined the effects of aroma inhalation on sleep quality, professional QoL, and near-misses in medication errors among nurses working in the emergency room on night duty. First, regarding the effect on sleep quality, the VSH sleep scale scores significantly improved on days 2 and 3 when aroma inhalation was applied compared with no treatment; this effect increased even more over time. The sleep duration increased on day 2 when aroma inhalation was applied compared to no treatment; the difference was not statistically significant. However, it significantly improved on day 3 after aroma inhalation compared to no treatment. These results are consistent with those of previous studies reporting the effectiveness of aroma inhalation on sleep quality [21,30]. Kim [30] reported that VSH sleep scale scores in the intervention group (51.45 ± 10.04) significantly increased after three days of aroma inhalation compared to the control group (41.39 ± 7.36) among nurses working fixed night shifts. This suggests that aroma inhalation effectively improves sleep quality among nurses working night shifts. Among the aroma oils used in this study, lavender and bergamot oils exhibit calming, muscle-relaxing, and anxiety-reducing effects, whereas the primary component of ylang-ylang oil, linalool, has a calming effect and influences sleep quality [39]. However, Kim & Hur [29] found that VSH sleep scale scores in the intervention group significantly increased on day 2 (47.87 ± 10.42) and day 3 (56.13 ± 10.87) compared to the control group (47.43 ± 9.46). This differs from the findings of our study, in which positive effects on sleep quality were observed on the first day after aroma inhalation. Furthermore, in a study by Oh et al. [40], where female production workers on rotating shifts inhaled bergamot, lavender, and ylang-ylang oils for four weeks, the VSH sleep scale scores in the intervention group (44.77 ± 8.11) significantly increased compared to the control group (43.12 ± 8.42). Despite the lower frequency of aroma inhalation sessions in our study, sleep quality improved earlier and to a greater extent than in studies by Kim and Hur [29] and Oh et al. [40]. These results are similar to those of Oh et al. [39], who observed psychological and physiological changes following aroma inhalation after just 1-2 sessions.

In this study, sleep duration following aroma inhalation increased by 30 min on day 2 compared with the control condition, although this difference was not statistically significant. On day 3, aroma inhalation was associated with a 50-minute increase in sleep duration relative to the control condition. However, despite these improvements, the total sleep time on both days remained shorter than the baseline sleep duration before the night shift. This suggests a potential cumulative effect of aroma inhalation on sleep duration over consecutive night shifts, warranting further investigation of its long-term efficacy in improving sleep patterns among night-shift nurses. Contrarily, Chang et al. [35] examined the effects of lavender, lemon, and sandalwood oil-infused necklaces, twice a day for two days, on sleep duration in night-shift nurses. After the first aroma inhalation session, their study found an increase of 6 minutes in sleep time (364.29 ± 90.79) compared to no treatment (358.24 ± 121.34). However, after the second aroma inhalation session, sleep time decreased (316.47 ± 61.17), with no statistically significant difference. This may be attributed to the fact that we used lavender, bergamot, and ylang-ylang oils, which have calming and relaxing effects. However, Chang et al. [35] used lemon oil, which stimulates the brain and has awakening effects [39]. Thus, the improved sleep quality and increased sleep time observed from the first day of application in our study, compared to previous research, might be attributed to the significant calming effects of lavender, bergamot, and ylang-ylang oils. Kim et al. [21] highlighted that combining lavender with other essential oils known for their relaxation and calming effects significantly enhances sleep quality. This enhanced efficacy is believed to result from the synergistic pharmacological effects of blending lavender with multiple oils. Additionally, selecting appropriate essential oils is crucial for designing aromatherapeutic interventions for sleep studies. Some aromatic oils activate the sympathetic nervous system and stimulate β-waves in brain activity, promoting alertness and arousal. These stimulatory effects counteract the intended sleep-inducing benefits of aromatherapy. Therefore, carefully considering the specific properties of each essential oil and choosing those that align with the study’s objectives is essential. It may be necessary to apply a combination of essential oils with calming effects rather than a single fragrance to effectively improve the sleep quality of nurses working night shifts.

Second, when considering the effects of aroma inhalation on professional QoL, compassion satisfaction improved after aroma inhalation compared to no treatment, but the difference was not statistically significant. Exhaustion and secondary traumatic stress, including compassion fatigue, also decreased after aroma inhalation compared to no treatment; however, the difference was not statistically significant. In a study by Shin et al. [28] involving emergency room nurses who inhaled patchouli oil for two days, compassion satisfaction significantly increased in the treatment group (34.00 ± 4.12) compared to the control group (30.48 ± 4.82), and compassion fatigue decreased in the treatment group (25.88 ± 4.63; control group: 26.48 ± 5.10), although the differences were not statistically significant. These discrepancies may be partly attributed to variations in the number of aroma inhalation sessions and the types of essential oils used. This study used a single session with a blend of bergamot, lavender, and ylang-ylang oils, whereas Shin et al. [28] used patchouli oil in two sessions. Although there is limited prior research on the effects of aroma inhalation on professional QoL, increased empathy satisfaction has been shown to lead to reduced compassion fatigue [3], potentially enhancing professional QoL. Therefore, further studies are required to confirm the effects of increasing the frequency of aroma inhalation on compassion satisfaction and reducing compassion fatigue. Stopen [41], who targeted emergency room nurses and applied compassion fatigue prevention education and aroma inhalation for eight weeks, found significant improvements in compassion satisfaction (39.80) and secondary traumatic stress (22.40) after aroma inhalation compared to pre-intervention levels of compassion satisfaction (36.63) and secondary traumatic stress (26.08). Burnout (28.13) decreased, but the difference was not statistically significant. While directly comparing these results to those of our study may be challenging, since Stopen’s research [41] involved a combination of aroma inhalation and education, it is evident that long-term multifaceted interventions positively impacted the professional QoL of emergency room nurses. This highlights the need to develop and implement long-term intervention programs, including aromatherapy inhalation, to enhance the professional QoL of emergency room nurses [11].

Finally, when examining the effects on near-misses in medication errors, aroma inhalation in this study did not yield statistically significant effects. No previous studies have shown how aroma inhalation affects near-misses in medication errors; however, according to Kim et al. [13], medication errors are significantly related to sleep. Moreover, Chaiard et al. [42] reported that sleep durations shorter than 7 hours were associated with an increased incidence of medication errors. Stimpfel et al. [43] identified a correlation between disturbed sleep patterns and elevated error risk in healthcare settings. Our findings demonstrate that, despite improved sleep quality attributed to aroma inhalation, there was no corresponding decrease in medication near-misses during a single night shift. In the emergency department where this study was conducted, nurses were assigned to night shifts for only two consecutive days. As a result, aromatherapy intervention for medication near-misses was applied for a single night shift. Thus, we could not confirm the effects of reducing near-misses in medication errors through improvements in sleep quality. The frequency of near-misses in medication errors possibly increases with the number of night shifts worked by nurses, which, in turn, affects their concentration [13]. Therefore, to confirm the reduction of near-misses in medication errors through improved concentration with aroma inhalation during extended night shifts, it is necessary to select aromas such as lemon, basil, and peppermint [39], which have more direct effects on concentration improvement, and eucalyptus [21], which activates the sympathetic nervous system and induces alertness during prolonged night shift work.

Furthermore, experiences with near-misses in medication errors rely on participants’ memory and are measured through subjective responses, possibly influenced by psychological biases [44], limiting measurement accuracy. In this study, we utilized and adapted the tool developed by Park and Lee [37] to measure near-misses in medication errors. While the original tool measured near-misses in medication errors experienced over the past three months, our study measured near-misses occurring on the same day, resulting in less reliable outcomes. Therefore, to measure near-misses in medication errors accurately, measurement methods such as direct observation must be employed. Additionally, as this study relied on individual memory-based measures without accounting for workload and intensity during the night shift, future replication studies should consider adjusting for these factors.

Melnyk et al. [45] found a negative correlation between nurses’ well-being and the likelihood of medical errors. Future research should explore the potential impact of enhancing the professional QoL on reducing medication near-misses. Such studies could investigate the efficacy of interventions, including patchouli oil aromatherapy, in improving nurses’ professional QoL and, by extension, patient safety outcomes. This study’s scope was limited to nurses working in the emergency department of a single tertiary care hospital, limiting the generalizability of the findings to a broader nursing population.

## CONCLUSION

Aroma inhalation improved the sleep quality of emergency room nurses working night shifts. These findings suggest the potential utility of aroma inhalation as an intervention to enhance sleep quality among shift workers, including nurses. The employed randomized crossover design allowed participants to experience both aroma inhalation and no treatment across two night shifts. This facilitated a more precise evaluation of the effects of aroma inhalation by ensuring balanced exposure, minimizing the influence of individual differences among the participants, and controlling for period effects. Additionally, given the limited research on the effects of aroma inhalation on professional QoL and near-misses in medication errors among emergency room nurses, this study is significant in exploring various outcome measures related to the effects of aroma inhalation.

## Figures and Tables

**Table 1.** General Characteristics of Participants (N = 55)

Variables	Characteristics	n (%)			χ^2^ or t	*p*
		Group 1	Group 2	Total		
		(n = 29)	(n = 26)			
Sex	Women	25 (86.2)	21 (80.8)	46 (83.6)	0.30	.586
	Men	4 (13.8)	5 (19.2)	9 (16.4)		
Age (yr)	< 25	6 (20.7)	2 (7.7)	8 (14.5)	4.57	.335
	25~29	15 (51.8)	18 (69.2)	33 (60.0)		
	30~34	5 (17.2)	4 (15.4)	9 (16.4)		
	35~39	2 (6.9)	0 (0)	2 (3.6)		
	≥ 40	1 (3.4)	2 (7.7)	3 (5.5)		
	M ± SD	28.14 ± 4.81	28.69 ± 4.43	28.40 ± 4.60	−0.44	0.661
Marital status	Married	6 (20.7)	3 (11.5)	9 (16.4)	0.84	.360
	Unmarried	23 (79.3)	23 (88.5)	46 (83.6)		
Education level	Diploma	3 (10.3)	4 (15.4)	7 (12.7)	2.93	.231
	Bachelor	25 (86.3)	18 (69.2)	43 (78.2)		
	≥ Master	1 (3.4)	4 (15.4)	5 (9.1)		
Total clinical career (yr)	< 2	6 (20.7)	5 (19.2)	11 (20.0)	0.46	.927
	2~ < 5	13 (44.8)	10 (38.5)	23 (41.8)		
	5~ < 10	7 (24.2)	7 (26.9)	14 (25.5)		
	≥ 10	3 (10.3)	4 (15.4)	7 (12.7)		
	M ± SD	67.10 ± 61.89	64.92 ± 53.25	66.07 ± 55.45	0.14	.889
Clinical career at emergency room (yr)	< 2	8 (27.6)	8 (30.8)	16 (29.0)	0.56	.905
	2~ < 5	14 (48.3)	11 (42.3)	25 (45.5)		
	5~ < 10	5 (17.2)	5 (19.2)	10 (18.2)		
	≥ 10	2 (6.9)	2 (7.7)	4 (7.3)		
	M ± SD	51.48 ± 43.16	45.31 ± 39.43	48.56 ± 41.17	0.55	.582
Religion	None	24 (82.9)	21 (80.8)	45 (81.8)	0.57	.903
	Protestantism	3 (10.3)	2 (7.7)	5 (9.1)		
	Catholicism	1 (3.4)	1 (3.8)	2 (3.6)		
	Buddhism	1 (3.4)	2 (7.7)	3 (5.5)		
Living with	Alone	12 (41.4)	13 (50.0)	25 (45.5)	0.41	.522
	With family	17 (58.6)	13 (50.0)	30 (54.5)		
Health status	Excellent	5 (17.2)	0 (0)	5 (9.1)	6.28	.099
	Very good	7 (24.2)	11 (42.3)	18 (32.7)		
	Good	15 (51.7)	12 (46.2)	27 (49.1)		
	Fair	2 (6.9)	3 (11.5)	5 (9.1)		
	Poor	0 (0)	0 (0)	0 (0)		
Presence of an underlying disease	No	25 (86.2)	23 (88.5)	48 (87.3)	0.06	.802
	Yes	4 (13.8)	3 (11.5)	7 (12.7)		
Taking medication	No	26 (89.7)	23 (88.5)	49 (89.1)	0.31	.576
	Yes	3 (10.3)	3 (11.5)	6 (10.9)		
Exercise	No	8 (27.6)	3 (11.5)	11 (20.0)	4.52	.340
	1~2 times/week	9 (31.0)	11 (42.3)	20 (36.4)		
	3~4 times/week	7 (24.2)	10 (38.5)	17 (30.9)		
	5~6 times/week	4 (13.8)	2 (7.7)	6 (10.9)		
	Every day	1 (3.4)	0 (0)	1 (1.8)		
Caffeine	No	4 (13.8)	4 (15.4)	8 (14.5)	5.20	.268
	1 cup/week	4 (13.8)	3 (11.5)	7 (12.7)		
	1 cup/2~3 days	6 (20.7)	10 (38.5)	16 (29.1)		
	1~2 cups/day	11 (37.9)	9 (34.6)	20 (36.4)		
	3 cups/day	4 (13.8)	0 (0)	4 (7.3)		
Alcohol	No	9 (31.0)	8 (30.8)	17 (30.9)	1.40	.706
	1~2 times/week	18 (62.2)	14 (53.9)	32 (58.2)		
	3~4 times/week	1 (3.4)	3 (11.5)	4 (7.3)		
	5~6 times/week	1 (3.4)	1 (3.8)	2 (3.6)		

Group 1 received aromatherapy treatment first, followed by no treatment, Group 2 received no treatment first, followed by aromatherapy treatment. M = Mean; SD = Standard deviation.

**Table 2.** Homogeneity Test of Dependent Variables between the Two Groups (N = 55)

Variables		Period	Group 1	Group 2	Linear mixed-effects model		
			M ± SD	M ± SD	Source	t	*p*
Quality of sleep		Period 1	47.72 ± 15.68	44.88 ± 11.80	Group	0.00	.998
		Period 2	48.14 ± 18.16	50.96 ± 15.50	Period	1.65	.103
		Total	47.93 ± 16.82	47.92 ± 13.97			
Sleep time		Period 1	7.19 ± 3.10	6.92 ± 2.48	Group	−0.32	.748
		Period 2	7.09 ± 3.27	7.81 ± 2.51	Period	1.10	.276
		Total	7.14 ± 3.16	7.37 ± 2.51			
Professional quality of life	Compassion satisfaction	Period 1	30.69 ± 5.36	30.38 ± 7.01	Group	0.72	.478
		Period 2	31.28 ± 4.53	29.31 ± 7.27	Period	−0.47	.639
		Total	30.98 ± 4.93	29.85 ± 7.07			
	Compassion fatigue	Period 1	55.93 ± 5.98	57.69 ± 7.19	Group	−1.19	.238
		Period 2	55.38 ± 7.45	57.85 ± 7.32	Period	−0.33	.740
		Total	55.66 ± 6.70	57.77 ± 7.19			
Near-misses in medication errors		Period 1	0.00 ± 0.00	0.19 ± 0.63	Group	−1.62	.110
		Period 2	0.00 ± 0.00	0.00 ± 0.00	Period	−1.54	.130
		Total	0.00 ± 0.00	0.10 ± 0.45			

M = Mean; SD = Standard deviation.

**Table 3.** Differences in Quality of Sleep, Sleep Time, Professional Quality of Life, and Near-Misses in Medication Error between Two Groups (N = 55)

Variables		Group	Aroma Tx	Non Tx	Linear mixed effects model				
			M ± SD	M ± SD		Df	t	*p*	95% CI
Quality of sleep at day 2		1	49.95 ± 12.89	43.71 ± 15.51	Group	53	0.43	.670	
		2	48.54 ± 13.82	42.42 ± 13.02	Period	53	−0.03	.975	
		Total	49.28 ± 13.23	43.10 ± 14.27	Treat	53	3.04	.004	2.10~10.26
Quality of sleep at day 3		1	54.48 ± 14.83	47.48 ± 13.07	Group	53	0.35	.727	
		2	53.15 ± 11.74	46.69 ± 12.39	Period	53	−0.15	.885	
		Total	53.85 ± 13.35	47.11 ± 12.64	Treat	53	3.64	.001	3.03~10.43
Sleep time at day 2		1	6.60 ± 1.09	6.43 ± 1.90	Group	53	−0.32	.750	
		2	6.67 ± 1.28	6.56 ± 1.34	Period	53	−0.12	.906	
		Total	6.64 ± 1.72	6.49 ± 1.65	Treat	53	0.60	.552	−0.34~0.63
Sleep time at day 3		1	6.66 ± 2.09	5.89 ± 1.71	Group	53	−0.27	.791	
		2	6.77 ± 1.56	5.98 ± 1.63	Period	53	0.04	.968	
		Total	6.71 ± 1.84	5.93 ± 1.66	Treat	53	2.76	.008	0.21~1.34
Professional quality of life	Compassion satisfaction	1	30.62 ± 4.76	30.07 ± 4.68	Group	53	−0.04	.970	
		2	30.58 ± 7.15	30.23 ± 7.20	Period	53	−0.23	.821	
		Total	30.60 ± 5.95	30.15 ± 5.95	Treat	53	0.99	.326	−0.46~1.36
	Compassion fatigue	1	55.00 ± 7.49	55.00 ± 6.77	Group	53	−1.10	.274	
		2	56.23 ± 7.34	57.88 ± 7.27	Period	53	−1.44	.156	
		Total	55.58 ± 7.38	56.36 ± 7.10	Treat	53	−1.44	.156	−1.98~0.33
Near-misses in medication errors		1	0.00 ± 0.00	0.00 ± 0.00	Group	53	−1.79	.080	
		2	0.00 ± 0.00	0.15 ± 0.46	Period	53	−1.79	.080	
		Total	0.00 ± 0.00	0.07 ± 0.33	Treat	53	−1.79	.080	−0.16~0.01

Tx = Treatment; M = Mean; SD = Standard deviation; Df = Degrees of freedom; CI = Confidence interval.

## Data Availability

The data that supports the findings of this study are available from the corresponding author upon reasonable request.
